# Sarecycline treatment for acne vulgaris: Rationale for weight‐based dosing and limited impact of food intake on clinical efficacy

**DOI:** 10.1111/dth.15275

**Published:** 2021-12-30

**Authors:** Ayman Grada, James Q. Del Rosso, Emmy Graber, Christopher G. Bunick, Linda Stein Gold, Angela Y. Moore, Hilary Baldwin, Zaidal Obagi, Giovanni Damiani, Timothy Carrothers, Brian McNamee, Eva Hanze

**Affiliations:** ^1^ R&D and Medical Affairs Almirall US Malvern Pennsylvania USA; ^2^ JDR Dermatology Research/Thomas Dermatology Nevada USA; ^3^ Dermatology Institute of Boston Boston Massachusetts USA; ^4^ Northeastern University Boston Massachusetts USA; ^5^ Department of Dermatology Yale University School of Medicine New Haven Connecticut USA; ^6^ Dermatology Clinical Research Henry Ford Health System Detroit Michigan USA; ^7^ Arlington Center for Dermatology Arlington Texas USA; ^8^ Acne Treatment and Research Center Morristown New Jersey USA; ^9^ Department of Dermatology University of Arizona Tucson Arizona USA; ^10^ Clinical Dermatology IRCCS Galeazzi Orthopaedic Institute Milan Italy; ^11^ Pharmacometrics AbbVie (formerly Allergan) Pleasanton California USA; ^12^ Clinical Pharmacology AbbVie (formerly Allergan) Dublin Ireland; ^13^ Pharmacometrics qPharmetra Stockholm Sweden

**Keywords:** absorption, acne, antibiotics, bioavailability, diet, dosing, efficacy, pharmacokinetics

## Abstract

Tetracycline‐class antibiotics are frequently prescribed by dermatologists, commonly for acne vulgaris. Gastrointestinal absorption of first and second‐generation tetracycline‐class antibiotics, including doxycycline and minocycline, may be reduced by co‐administration with food, resulting in potentially lower clinical efficacy. Development of novel compounds and formulations that are not impacted by diet could improve compliance, absorption, and effectiveness among patients. The objective of this study is to investigate weight‐based dosing protocols and the impact of food intake, including high‐fat meals, on the absorption, and clinical efficacy of sarecycline, a novel oral narrow‐spectrum third‐generation tetracycline‐class antibiotic approved by the Food and Drug Administration for acne vulgaris treatment. Data from 12 clinical studies were analyzed using population pharmacokinetic modeling, exposure–response modeling and pharmacodynamics to evaluate sarecycline dosing recommendations. The extent of exposure is estimated to decrease by 21.7% following co‐administration of a sarecycline tablet with a high‐fat meal. Based on the PopPK‐PD model, this is equivalent to a decrease in efficacy of 0.9 inflammatory lesions, which is not clinically meaningful. Sarecycline can be administered using weight‐based dosing with or without food. Co‐administration with high‐fat food has a limited impact on clinical efficacy. The pharmacokinetics of oral sarecycline may provide added convenience and support ease of use and improved compliance for acne vulgaris patients.

## 1 INTRODUCTION

Tetracycline‐class antibiotics are frequently prescribed by dermatologists, primarily for the treatment of acne vulgaris and rosacea.^1^, ^2^ Tetracycline‐class antibiotics include tetracycline (first generation), doxycycline and minocycline (second generation), and sarecycline (third generation).^3^, ^4^ Prior to sarecycline, all tetracycline‐class antibiotics exhibited broad‐spectrum activity against a wide range of aerobic and anaerobic Gram‐positive and Gram‐negative bacteria.^3^ This broad‐spectrum activity may induce bacterial resistance and disrupt the microflora, leading to dysbiosis in the gastrointestinal (GI) and genitourinary (GU) tracts, which are associated with adverse events (AEs) such as diarrhea and vulvovaginal mycotic/candidiasis infections.^5^, ^6^, ^7^, ^8^ In addition, prolonged use of broad‐spectrum antibiotics such as doxycycline has been associated with an increased risk of inflammatory bowel disease^9^, ^10^, ^11^

Sarecycline is an immediate‐release tetracycline derivative, designed with a stable structural modification at hydrocarbon C7, to produce a narrow spectrum antibiotic profile inclusive of activity against *Cutibacterium acnes*.^3^ The structural modifications incorporated result in direct binding to the bacterial mRNA within the bacterial ribosome 30S subunit and a low propensity to induce antibiotic resistance.^3^, ^12^, ^13^ The rationale for an immediate‐release formulation of sarecycline is supported by minimal GI‐associated AEs noted in clinical studies.^3^, ^12^, ^13^, ^14^, ^15^ Sarecycline has demonstrated activity against clinically relevant Gram‐positive bacteria, and reduced activity against Gram‐negative bacteria commonly found in the GI tract, with a low rates of AEs reported in pivotal phase 2 and phase 3 clinical trials and in an 40‐week safety extension study.^3^, ^14^, ^15^

Oral antibiotics may vary in their bioavailability depending on several factors including intra‐individual weight‐to‐dose ratio and potential food‐drug interactions. The existing tetracyclines‐class antibiotics, tetracycline, doxycycline and minocycline, are structurally bound by diets and medications that are high in divalent and trivalent metal ions such as calcium, magnesium, iron, and aluminum, which can reduce GI absorption of the antibiotics; iron has been shown to markedly reduce GI absorption of doxycycline and minocycline.^16^, ^17^, ^18^, ^19^ In addition, ingestion with a high‐fat meal may reduce doxycycline GI absorption. Following single dose administration of enteric‐coated delayed release 150‐mg doxycycline tablet, the mean maximum plasma concentration (*C*_max_) was 19% lower with a high fat meal, including milk, compared to fasted conditions.^19^ The effect from food can vary based on the dose (less effect at higher doses). However, the clinical impact has not been studied. Additionally, a decrease in the rate (*C*_max_) and extent (area‐under‐the curve [AUC]) of GI absorption of 45% and 22%, respectively, were reported in human subjects given a single oral dose of a brand doxycycline 40 mg modified‐release capsule after ingestion with a 1000 calorie, high fat, high protein meal that included dairy products when compared to fasted conditions.^20^ The FDA‐approved prescribing information with this doxycycline modified‐release formulation states that this decrease in systemic exposure may be clinically significant with the recommendation that it be taken at least 1 h prior to or 2 h after meals.^20^

Especially with immediate‐release formulations, patients are commonly instructed to ingest doxycycline with food to reduce GI side effects.^18^, ^21^, ^22^, ^23^ and a large volume of liquid (water). Administration of doxycycline taken without food and a large glass of water has been associated with “pill esophagitis” which causes pain, and gastric side effects such as nausea and vomiting.^19^, ^21^, ^22^ Likewise, ingestion of food along with minocycline is reported to help reduce the risk of esophageal irritation and ulceration.^18^ Lastly, oral tetracycline is currently suggested to be given 1–2 h before meals to reduce potential impact of food on GI absorption.^24^

Currently, sarecycline has been Food and Drug Administration (FDA)‐approved specifically for the treatment of moderate‐to‐severe acne vulgaris in patients 9 years of age and older, and can be taken once daily with or without food.^25^ The recommended dosage of sarecycline is based on body weight and is available in three daily dosages (60, 100, and 150 mg)^24^ (Table 1). Previous clinical and non‐clinical studies of sarecycline pharmacokinetics (PK) suggest that it may be administered with or without food, which supports this recommendation in the approved product labeling.^25^, ^26^, ^27^ The data evaluation below reports integrative PK/pharmacodynamics (PD) analyses evaluating both the relevance of weight‐based dosing and the impact of food intake on the efficacy of sarecycline.

**TABLE 1:** Recommended weight‐based dosing for sarecycline

Body weight (kg)	Tablet strength (mg)
33–54	60
55–84	100
85–136	150

## 2 METHODS

### 2.1 Data and software

Population pharmacokinetic (PPK) modeling and exposure–response (E‐R) modeling were utilized to support dosing recommendations. The analysis was carried out using NONMEM (Version 7.3, ICON Development Solutions) on workstations with Intel® Core™ i7 processors, Windows 7 Professional and the GNU gfortran compiler (Version 4.5.0, http://ftp.globomaxnm.com/Public/nonmem7/compilers). Post‐processing of NONMEM analysis results was carried out in R version 3.2.2. The stepwise covariate modeling (SCM) was carried out using Perl‐speaks‐Nonmem (PsN), version 4.2.0. Model development was carried out using first order conditional estimation with Interaction (FOCE‐I).

Initial development of PK/PD models was based on single‐ and multiple dose data inclusive of results in 144 healthy subjects. These patients were obtained from 12 clinical studies (Table 2). These PK/PD models were utilized to guide phase 3 study design. Upon completion of phase 3 studies, the PK/PD models were expanded to include the phase 3 study data with the results reported here.

**TABLE 2:** Summary of data included in population pharmacokinetic analysis

Study	Study design	Study population	*N*	*N* ^a^	Treatment
PR‐10711	Food effect of capsules	Healthy males and females	16	16	240 mg capsule, fasted or following high fat meal
PR‐01010	Placebo‐controlled single dose study	Healthy males	64	48	20–480 mg capsules, fasted
PR‐05011	Placebo‐controlled multiple dose study	Healthy males	56	42	40–320 mg QD for 7 days, capsules, fasted
PR‐07112	Thorough QT/QTc study	Healthy males and females	48	41	500 mg capsule, fasted
PR‐11914	Food effect and bioavailability for tablet formulation	Healthy males and females	19	19	150 mg tablet versus aqueous solution, fasted versus high‐fat meal
PR‐12014	Relative bioavailability of tablet and capsule formulations	Healthy males and females	26	26	100 mg capsule versus tablet, fasted
SRC‐PK‐03	Hepatic impairment study: Child‐Pugh A, B versus normal (8/group)	Healthy males and females	24	24	150 mg tablet, light meal 1–2 h predose
SRC‐PK‐04	Renal impairment study: Mild, moderate, or severe renal impairment versus normal (8/group)	Healthy males and females	32	32	150 mg tablet, light meal 1–2 h predose
SRC‐PK‐06	Multiple dose pharmacokinetic study of tablet formulation at three dose levels	Healthy males and females	24	24	60, 100, and 150 mg tablet QD, fasted for 7 days
SRC‐PK‐08	Drug–drug interaction to examine effect of sarecycline on oral contraceptive exposure	Healthy females	26	26	Oral contraceptive QD for 24 days + sarecycline 150 mg tablet QD, fasted
PR‐10411	Phase 2 study	Subjects with facial acne vulgaris	285	212	Placebo versus sarecycline capsules, 0.75, 1.5, or 3.0 mg/kg QD^b^
SC1401, SC1402	Phase 3 study	Subjects with facial acne vulgaris	~1000 each	~500 each	Placebo versus sarecycline, 1.5 tablet mg/kg QD^c^

Abbreviations: QD, once a day; mg, milligram.

^a^ Subjects treated with sarecycline.

^b^ Subjects received the same dose regardless of weight (50, 100, or 200 mg); weight range limited to 52–88 kg.

^c^ 1.5 mg/kg corresponds to 60 mg tablet for subjects who weighed 33–54 kg, 100 mg tablet for subjects who weighed 55–84 kg, and 150 mg tablets for subjects who weighed 85–136 kg.

### 2.2 Population pharmacokinetic modeling

**FIGURE 1:**
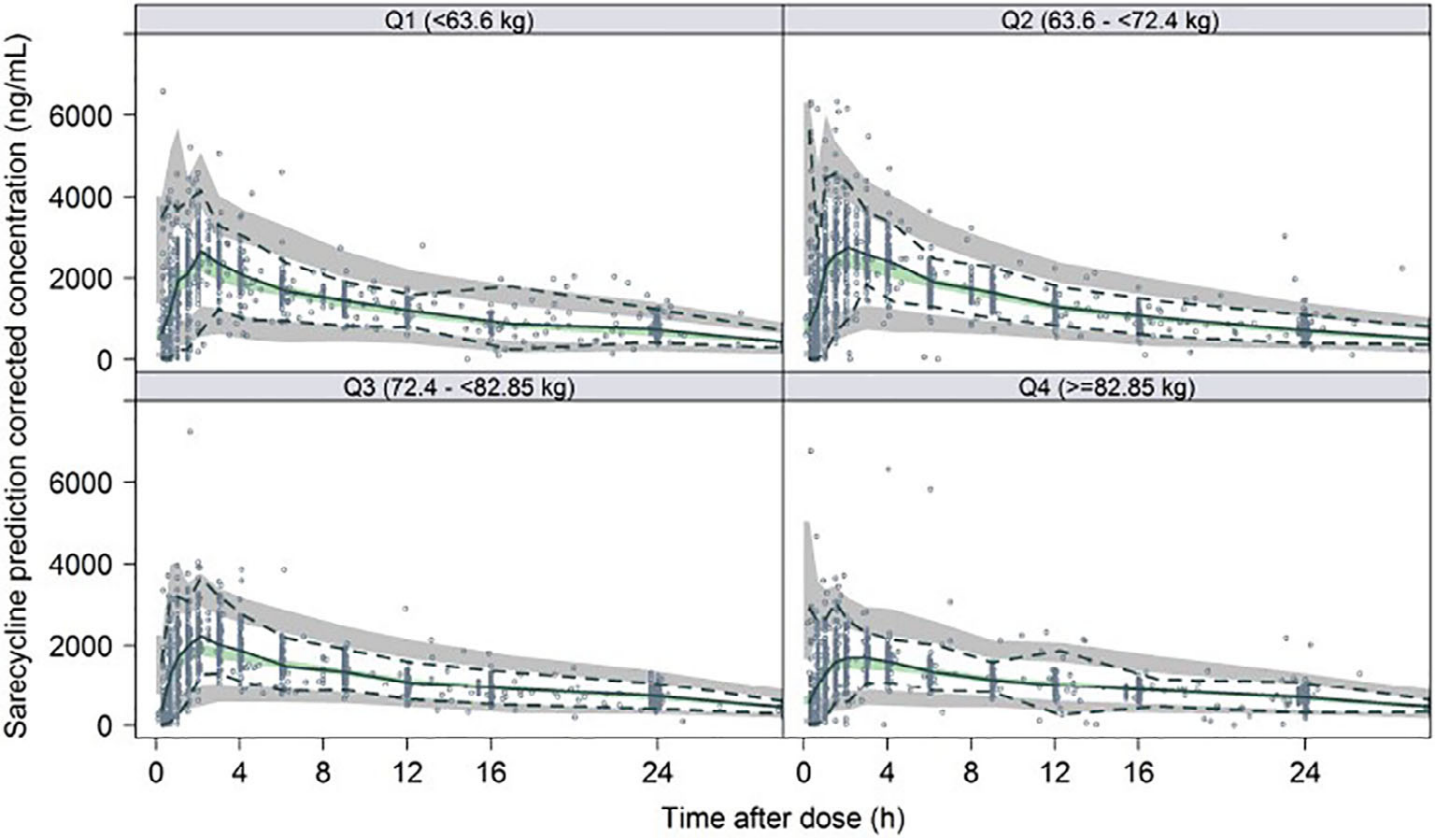
Visual predictive check for PK model stratified by body weight. Circles represent observations. Solid blue line represents median of the observed sarecycline concentrations. Dashed lines represented 2.5th and 97.5th percentiles of the observed sarecycline concentrations. Shaded areas represent the 95% confidence interval around the prediction‐corrected median (green area), and the 2.5th and 97.5th percentile of the simulated concentrations (gray areas). All observations and predictions are adjusted using prediction correction as described in Bergstrand et al.^23^

The structural pharmacokinetic (PK) model was initially developed based on phase 1 data with capsule and tablet formulations and was characterized using nonlinear mixed effects modeling. In brief, PPK modeling included estimated concentration time profiles at the patient level and provided for quantitative understanding of sarecycline plasma concentrations in fed/fasted states and according to body weight and gender. In the current analysis, PK data from subjects in two phase 3 pivotal studies were used to refine the structural PK model and included tablet formulations. The prediction corrected visual predictive check (VPC) normalizes observations and predictions from different dose groups to assess model fitness when not stratifying by dose (Figure 1). The central tendency of the data and variability are well‐described by the model. The parameters of the final PK model are found in Table S1.

### 2.3 Exposure–response modeling

**FIGURE 2:**
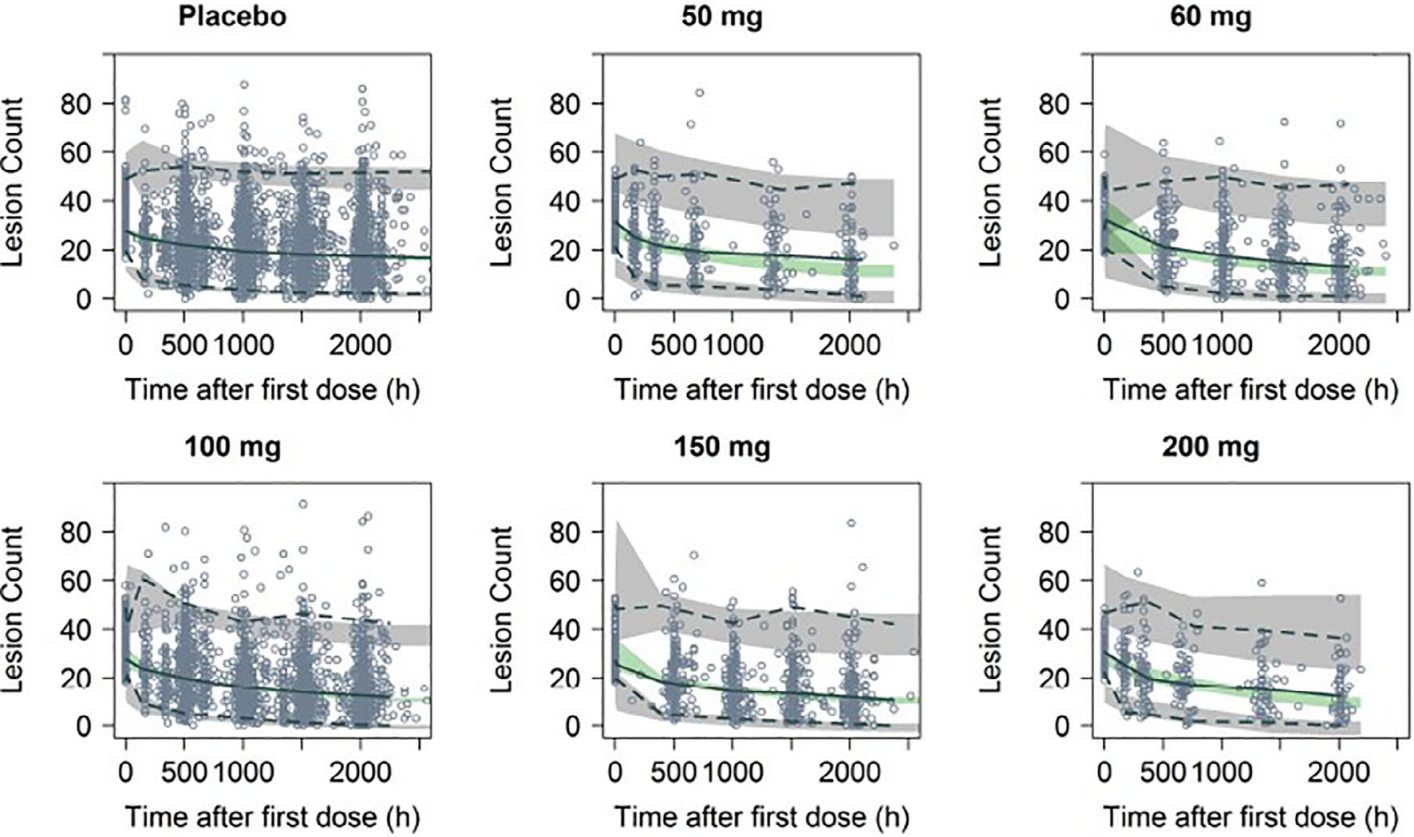
Visual predictive check of inflammatory lesion counts with the E‐R model. Circles represent observed concentrations. Solid black line represents median of the observed sarecycline concentrations. Dashed lines represented 2.5th and 97.5th percentiles of the observed concentrations. Shaded areas represent the 95% confidence interval around the prediction‐corrected median (green area), and the 2.5th and 97.5th percentile of the simulated concentrations (gray areas). All observations and predictions are adjusted using prediction correction as described in Bergstrand et al.^28^

The co‐primary endpoints in the phase 2 and the phase 3 studies were the absolute change from baseline in the facial inflammatory lesion counts at Week 12 and a dichotomized Investigator's Global Assessment (IGA) score (either “success,” denoted as ≥2‐point decrease from baseline in the IGA assessment and a score of clear [0] or almost clear [1], or “failure”) at Week 12. The E‐R model for inflammatory lesion count was a longitudinal model composed of placebo, time, baseline count, and drug effects. The “drug effect” portion quantified the relationship between different levels of exposure (area under the concentration‐time curve [AUC]) to differences in efficacy. Additionally, for drug effect, indirect response models were considered to account for time delay between sarecycline exposure and the associated observation of the endpoint. The E‐R analysis evaluated metrics of exposure (AUC at steady state [AUC_ss_]) and other subject characteristics, including gender, weight and age to inflammatory lesions counts and IGA. Individual sarecycline AUC_ss_ was derived using the PPK model. The VPC for inflammatory lesion counts shows that overall, the model reasonably predicts the inflammatory lesion counts versus time profile, although there is an over‐prediction trend of decrease in inflammatory lesion counts at later time‐points throughout all dose levels, including with placebo (Figure 2).

### 2.4 Simulations

Using the final PPK model, a simulation of the PK model was made using a uniform weight distribution to illustrate the impact of weight and dosing regimens without assuming any distribution of weight. Both the phase 3 dose regimen (weight‐based dosing wherein 60, 100, or 150 mg once daily was given for subjects who weighed 33–54, 55–84, or 85–136 kg, respectively) and a 100 mg dose regimen to all were simulated.

## 3 RESULTS

### 3.1 Weight‐based dosing

**FIGURE 3:**
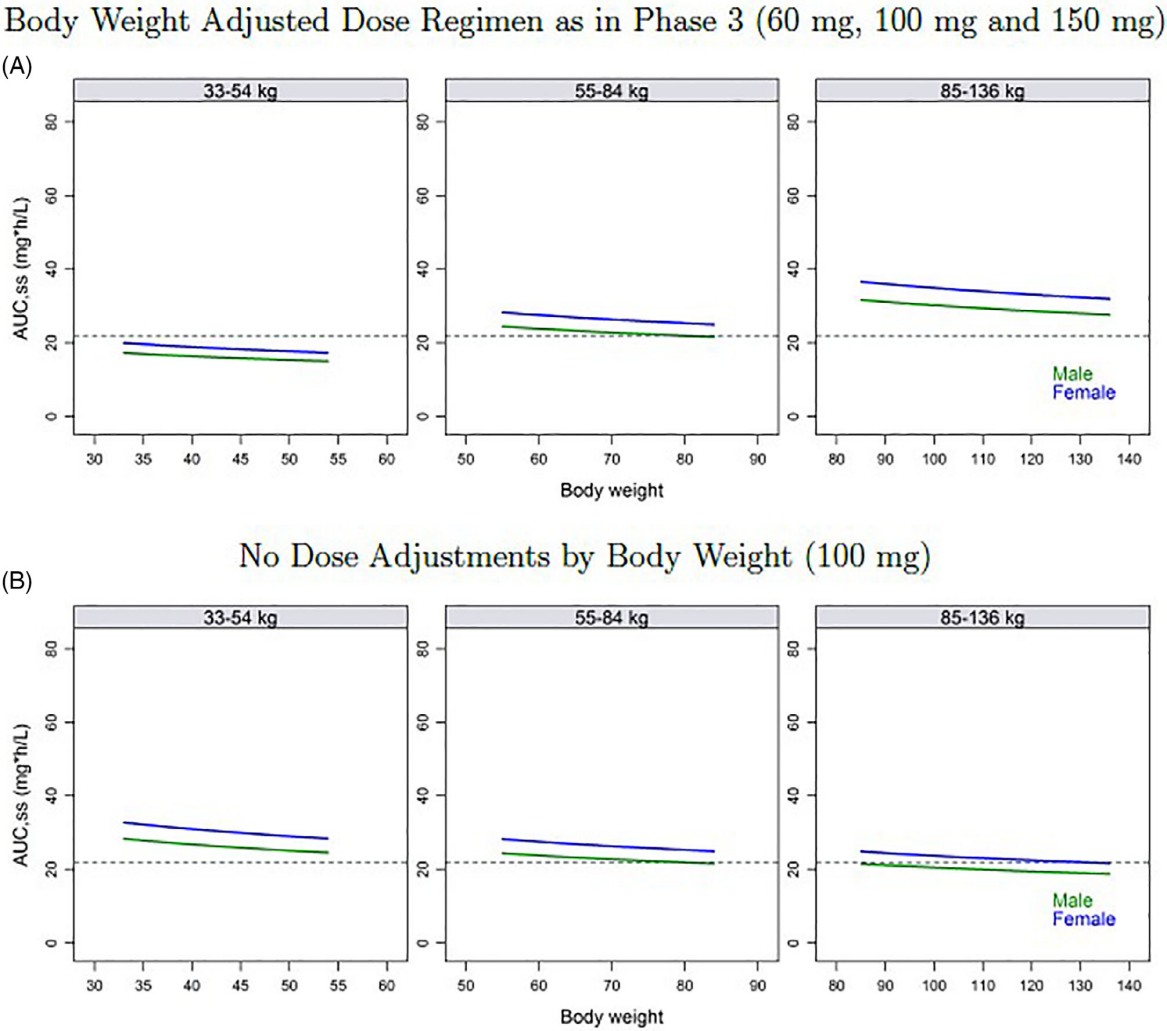
Model predicted AUC_ss_ using a uniform body weight distribution, by body weight category. (A) Body weight adjusted dose regimen as in Phase 3 (60 mg, 100 mg, and 150 mg). (B) No dose adjustments by body weight (100 mg). Horizontal dotted lines are the mean population AUC_ss_ prediction in a male with a body weight of 78.7 kg

**FIGURE 4:**
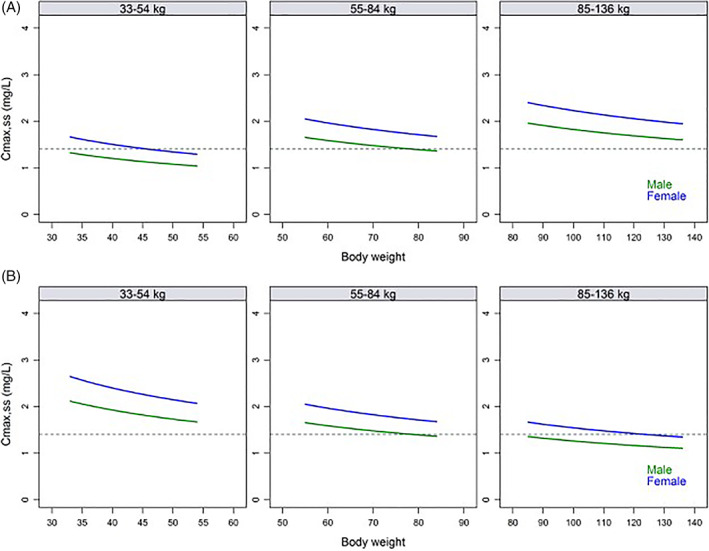
Model predicted *C*_max,ss_ using a uniform body weight distribution, by body weight category. (A) Body weight adjusted dose regimen as in Phase 3 (60 mg, 100 mg, and 150 mg). (B) No dose adjustments by body weight (100 mg). Horizontal dotted lines are the mean population *C*_max,ss_ prediction in a male with a body weight of 78.7 kg

A total of 562 subjects from 12 clinical studies (Table 2) were included in the PPK modeling. To note, subjects in phase 1 studies were healthy volunteers. PPK modeling was developed using a 2‐compartment model with first order absorption with a lag time and linear elimination (form the initial nonlinear mixed effects modeling). Weight effect was modeled by allometric scaling and with exponents estimates. Apparent clearance unadjusted for bioavailability (CL/F) was estimated to 3.15 L/h (relative standard error [RSE]: 2.11%), V_1_/F and apparent peripheral volume of distribution (V_2_/F) were estimated to 54.2 L (RSE: 2.63%) and 15.1 L (RSE: 6.48%), respectively and k_a_ was estimated to 3.45 h^−1^ (RSE: 16.0%). Prandial state and dose were predictors of both F and k_a_. The exponent of the normalized weight effect (CL/F_WT_) was estimated at 0.291. This simulation demonstrated that adjustment of dosing by body weight leads to similar differences in peak drug levels across weight groups compared with a 100 mg dose regimen (Table 3, Figures 3, 4).

**TABLE 3:** Summary of model predicted AUC_ss_ and *C*_max.ss_ at midpoint of each weight category using uniform body weight distribution

Dose regimen (mg)	Body weight (kg)	Dose (mg)	AUC_ss_ (mg h/L)	*C*_max,ss_ (mg/L)
60, 100, and 150	43	60	17.2	1.31
60, 100, and 150	70	100	24.6	1.65
60, 100, and 150	110	150	31.7	1.95
100	43	100	28.3	2.09
100	70	100	24.6	1.65
100	110	100	21.5	1.34

Abbreviations: AUC_ss_, area under the concentration versus time curve at steady state; C_max.ss_, maximum concentration at steady state.

### 3.2 Limited impact of food on sarecycline efficacy

**FIGURE 5:**
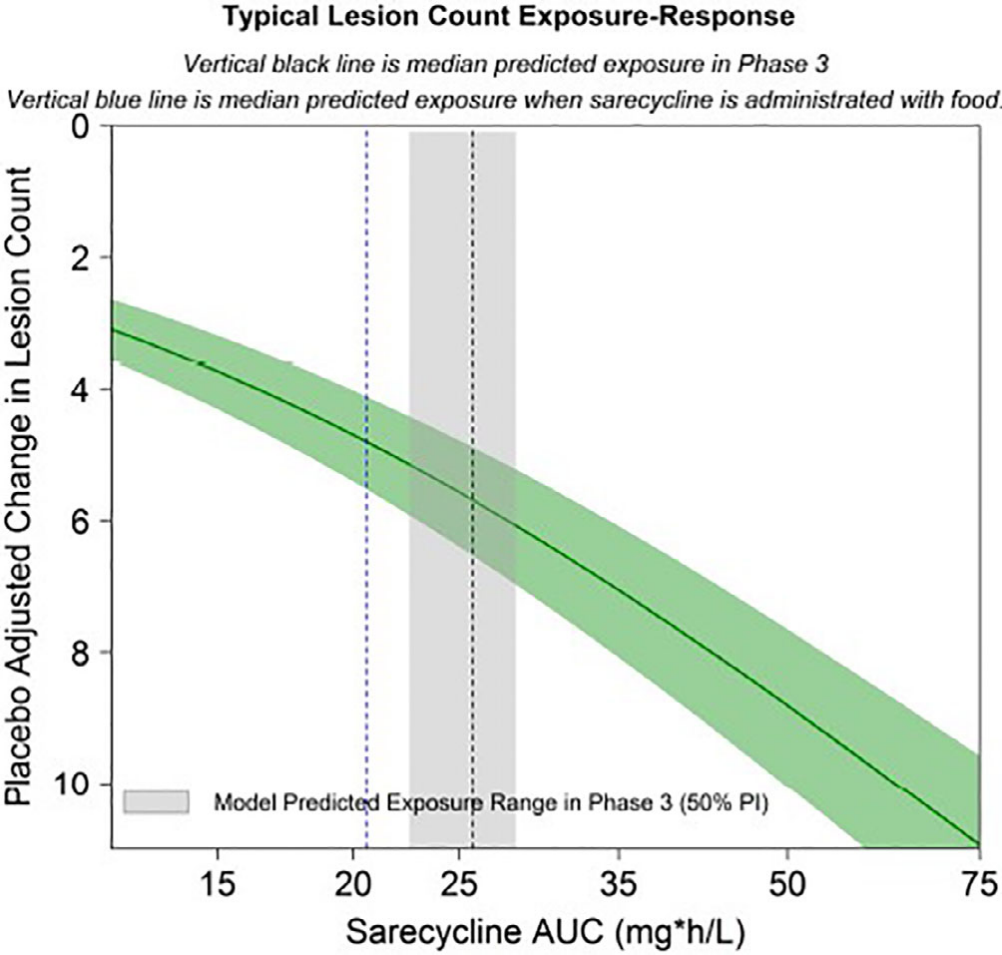
Model predicted typical inflammatory lesion counts exposure‐response plot. Gray area is 50% prediction interval of sarecycline AUC_ss_ in the phase 3 studies. Green area is 95% CI for the typical E‐R at 12 weeks. Vertical black line is median predicted exposure in Phase 3. Vertical blue line is median predicted exposure when sarecycline is administered with food. The net impacts on efficacy were not clinically significant and do not necessitate dose adjustments

The E‐R model examined inflammatory lesion counts with time, drug exposure, and placebo effects and other subject characteristics accounted for. Inflammatory lesion counts over 12 weeks was considered a continuous variable and was assessed using an indirect response model with the drug effect included on the production of lesion counts. In addition, the relationship between IGA response and sarecycline exposure (AUC_ss_) and other subject characteristics were evaluated using logistic regression methods. A combined simulation of PK and E‐R models indicates support for administration of sarecycline regardless of food intake. The exposure efficacy relationship was relatively flat over the range of concentrations seen in phase 3 subjects (Table 3, Figure 3A). Co‐administration of sarecycline with a high‐fat meal was estimated to result in a 21.7% reduction in exposure at steady state (AUC_ss_) and a reduced response of 0.9 inflammatory lesions (5.1 and 6.0 placebo adjusted change in lesion counts, respectively); this lesion count reduction is not clinically relevant (Figure 5).

## 4 DISCUSSION

The results of the current PK studies demonstrate that sarecycline can be administered using a weight‐based dosing regimen and can be taken with or without food without a clinical relevant impact on efficacy. As described above, data with older first‐ and second‐generation tetracycline‐class antibiotics report diet‐related effects, which may potentially have impact on efficacy in some patients. Co‐administration of a delayed‐release doxycycline formulation with a high‐fat meal reduced the overall bioavailability (AUC) by 15%–18% compared to a fasted state, with published reports stating reduction in doxycycline bioavailability by 26% and tetracycline bioavailability by 46%.^23^, ^29^ Given that patients who take doxycycline are commonly instructed to ingest the drug with food (including dairy products) to prevent GI tract‐associated AEs, it is difficult to assess the true clinical impact of this decrease in bioavailability, especially when lower doses are prescribed. Similarly, coadministration of minocycline with iron or food decreases its bioavailability by 77% and 13%–14%, respectively.^29^, ^30^

By contrast, PK/PD studies, coupled with PPK and E‐R model analyses, support that sarecycline is efficacious regardless of whether it is taken with or without food. In the current E‐R model, co‐administration of sarecycline with a high‐fat meal did not elicit a significant change in exposure or in clinically meaningful effects on inflammatory lesion counts, with a negligible inflammatory lesion decrease of 0.9 lesions. Additionally, analysis of simulation data of the PK model supports weight‐based dosing.

Based on bioavailbility clinical study (PR‐11914), co‐administration with a high‐fat (approximately 50% of total caloric content of the meal), high‐calorie (800–1000 kcal) meal that included milk produced a delay of *T*_max_ by 0.53 h, and decreases in *C*_max_ and AUC by 31% and 27%, respectively; these results deemed not to be clinically relevant.^25^, ^27^ Sarecycline is rapidly absorbed with a median time peak plasma concentration (*T*_max_) of 1.5–2 h and there are no significant differences in PK of sarecycline based on age, weight, gender, renal impairment, or mild to moderate hepatic impairment (Child Pugh A or B).^26^ Sarecycline bioavailability has not been evaluated in end‐stage renal disease or severe hepatic impairment (Child‐Pugh C). It is important to note that although the effect of food on sarecycline PK is comparable to doxycycline, the impact on sarecycline clinical efficacy, as shown in this study, was not clinically meaningful. No data exist for other tetracycline‐class drugs.

Weight‐based dosing allows for similar differences in plasma sarecycline concentration across weight groups when compared with a 100 mg dose regimen. Taken together, thorough analyses of data on weight‐based dosing and the efficacy of sarecycline in both fed and non‐fed states support recommendations in the FDA‐approved product labeling stating that oral sarecycline may be administered once daily with or without food, and dosed based on patient weight. Approval by the FDA for use in patients 9 years of age and older and data from pivotal phase 3 studies in patients with moderate to severe inflammatory acne vulgaris support the therapeutic and safety benefits of oral sarecycline in many patients.^25^ Given the additional benefit that sarecycline is administered daily with or without food, the convenience provided for the patient and the clinician are both highly favorable.

## CONFLICT OF INTEREST

Emmy Graber: honoraria and/or grants from Almirall, Allergan, Hovione, Sebacia, Alcimed, WebMD, WoltersKluwer, 3Derm, and Oakland Innovation. James Del Rosso: consultant and/or speaker (honoraria), and/or reserach investigator (grants) for Almirall, BioparmX, Bausch Health (Bausch Health), EPI Health, Galderma, JEM Health, LaRoche‐Posay, Leo Pharma, Main Pharma, Sebacia, SolGel, Sun Pharma, and Vyne Therapeutics (Foamix). Linda Stein Gold: honoraria and/or grants from Almirall, Bausch Health (Ortho Dermatologics), Cassiopeia, Galderma, Mayne Pharma, Sol Gel, Sun Pharma, and Vyne Therapeutics (Foamix). Angela Moore: receives funds as an advisory board member (A), consultant (C), clinical study investigator (I), and speaker (SP) for Almirall (C,I,SP), EpiHealth (A), Galderma (I), Mayne Pharma (C,I), Vyne (I,SP). Zaidal Obagi: no conflicts of interest. Hilary Baldwin: honoraria from Almirall, Bausch Health (Ortho Dermatologics), EPI Health, Galderma, LaRoche‐Posay, Mayne Pharma, EPI Health, Journey and SolGel. Timothy Carrothers: prior employee of Allergan with direct contribution to manuscript preparation, currently an employee of AbbVie, and owns stock in AbbVie. Brian McNamee: an employee of AbbVie and owns stock in AbbVie. Eva Hanze: no conflicts of interest. Giovanni Damiani: no conflict of interest. Christopher Bunick: honoraria and grants from Almirall. Ayman Grada; Head of R&D and Medical Affairs at Almirall US.

## AUTHOR CONTRIBUTIONS

Ayman Grada, Timothy Carrothers, Brian McNamee, and Eva Hanze, contributed to the conception and design, acquisition of data, analysis and interpretation of data, and drafting the manuscript. James Q. Del Rosso, Giovanni Damiani, Emmy Graber, Christopher G. Bunick, Linda Stein Gold, Angela Y. Moore, Hilary Baldwin, and Zaidal Obagi were involved in drafting the manuscript and revising it critically for important intellectual content.

## Supporting information


**Table S1** Parameters of Final Pharmacokinetic Model

## Data Availability

The data that support the findings of this study are available from the corresponding author upon reasonable request.
