# Supplementary material for: Sarecycline treatment for acne vulgaris: Rationale for weight‐based dosing and limited impact of food intake on clinical efficacy
Source: Dermatol Ther. 2021 Dec 30;35(3):e15275. doi: 10.1111/dth.15275 (PMC9286649; doi:10.1111/dth.15275)
Supplement: Supplementary file 1 — Table S1 Parameters of Final Pharmacokinetic Model [file DTH-35-0-s001.docx]

**Supplementary Tables**

**Table S1.** Parameters of Final Pharmacokinetic Model

| **PK model parameter** | **Covariates** |
| --- | --- |
| CL/F | Weight*, gender, age, renal and/or hepatic impairment status |
| F | Prandial status*, absolute dose, drug formulation (capsule vs. tablet) and number of capsules |
| V_n_/F | Weight*, gender, age |
| k_a_ | Age, prandial status*, absolute dose, drug formulation (capsule vs. tablet) and number of capsules |

*Weight and prandial status were included prior to the step-wise covariate search
